# Research on Abrasive Water Jet Polishing of Silicon Carbide Based on Fluid Self-Excited Oscillation Pulse Characteristics

**DOI:** 10.3390/mi14040852

**Published:** 2023-04-14

**Authors:** Hong Zhang, Baochun Tao, Qianfa Deng, Chengqi Zhang, Binghai Lyu, Duc-Nam Nguyen

**Affiliations:** 1 Ultra-Precision Machining Center, Zhejiang University of Technology, Hangzhou 310023, China; 2 Key Laboratory of Special Equipment and Advanced Processing Technology of Ministry of Education, Zhejiang University of Technology, Hangzhou 310023, China; 3 Special Equipment Institute, Hangzhou Vocational & Technical College, Hangzhou 310018, China; 4 School of Mechanical Engineering, Industrial University of Ho Chi Minh City, Ho Chi Minh City 800010, Vietnam

**Keywords:** plishing, silicon carbide, abrasive water jet, fluid self-excited oscillation

## Abstract

A self-excited oscillating pulsed abrasive water jet polishing method is proposed to solve the problems of low removal efficiency in traditional abrasive water jet polishing and the influence of an external flow field on the material surface removal rate. The self-excited oscillating chamber of the nozzle was used to generate pulsed water jets to reduce the impact of the jet stagnation zone on material surface removal and increase the jet speed to improve processing efficiency. ANSYS Fluent was employed to simulate the processing flow field characteristics for different lengths of oscillation cavities. The simulation results indicate that the velocity of the jet shaft reached a maximum of 178.26 m/s when the length of the oscillation cavity was 4 mm. The erosion rate of the material is linear with the processing angle. A nozzle with a length of 4 mm of the self-excited oscillating cavity was fabricated for SiC surface polishing experiments. The results were compared with those of ordinary abrasive water jet polishing. The experimental results showed that the self-excited oscillation pulse fluid enhanced the erosion ability of the abrasive water jet on the SiC surface and significantly improved the material-removal depth of the abrasive water jet polishing SiC. The maximum surface erosion depth can be increased by 26 μm.

## 1. Introduction

Silicon carbide materials have excellent physical, mechanical, and optical properties and have been widely used in optics, aerospace, semiconductors, and other fields [1,2,3,4,5]. However, its inherent properties, such as high hardness and brittleness, make it difficult to fabricate [6,7]. Therefore, researchers have proposed various processing methods, such as abrasive water jet processing [8], electric discharge machining [9], ultrasonic-assisted processing [10], and laser processing [11], to meet the high-efficiency and high-precision processing requirements of silicon carbide materials. Among these, abrasive water jet processing has attracted increasing attention because it lacks a heat-affected zone, small reaction force, wide processing range, and flexibility.

Zhu et al. [12] conducted polishing experiments on Si3N4 ceramic materials through an abrasive water jet (AWJ). The polishing experimental results showed that AWJ polishing significantly improved the surface of the workpieces. However, the level of surface quality achieved was affected by other factors, such as material properties and processing defects. Vijay [13] used the AWJ method to process a Ti-6Al-4 V alloy. The cut and material removal rate depths are higher at higher waterjet pressures. However, finer abrasives and lower water pressure are used to obtain better surface quality in abrasive water jet processing, and the processing efficiency is considerably low due to a stagnant layer [14]. Therefore, researchers have proposed various solutions to improve processing efficiency.

Qi et al. [15] introduced ultrasonic vibrations into a workpiece and performed polishing experiments on amorpgous glass. Studies have shown that the polishing efficiency of abrasive water jets significantly improves after the introduction of ultrasonic vibrations. Liu et al. [16] processed 4H-SiC wafers using vibration-assisted abrasive water jetting. They found that the applied vibration did not add energy to the impact process or affect the erosion depth of the particles. Nanoscale surface finishes were achieved, and material removal rates were improved.

Yu et al. [17] proposed a pneumatic-assisted abrasive water-jet processing technology. Based on the test results, the gas drive system vertically impacts the surface of the workpiece under the action of a 3 kg/cm^3^ air compression source. After 5 s, the surface of the workpiece formed a Gaussian removal pattern, and the removal depth was approximately 100 nm. In addition, compressed air increases the kinetic energy of the abrasive, making it more effective in removing the material.

Soyama et al. [18] used a high-speed submerged abrasive cavitation water jet to perform cavitation shot peening on titanium alloy Ti6Al4V samples prepared by direct laser sintering. Then, they carried out torsional fatigue tests. These results show that cavitation peening using a cavitation jet can extend the fatigue life of AMTi6Al4V by a factor of 1.4 when the applied stress is 450 MPa. Cavitation peening can increase the sample stiffness while suppressing cracks and other defects, thereby improving the fatigue performance of the sample. However, these methods require additional external energy to increase the total kinetic energy of the jet beam.

The fluid vibrates spontaneously in structures with defined boundaries [19], converting a continuous jet into a pulsed jet. Floddyna et al. [20] proposed a self-oscillating pulse jet polishing supported by ultrasonic waves. Rock-cutting experiments were conducted. Under the same experimental conditions, the obtained groove width and depth values were larger than those obtained continuously. Qianfa et al. [21] polished the inner wall of a stainless-steel capillary using self-excited oscillating water pulse grinding and spray polishing. After 12 h of treatment, the Ra of the inner tube wall dropped from 480 nm to 50 nm. The roughness decreased to 55 nm. Hui et al. [22] proposed a self-excited oscillating double-chamber abrasive water jet for polishing the inner walls of stainless-steel tubes. In the case of self-excited double-cavity oscillation, the surface roughness of the inner tube wall decreased from 452 nm to 42 nm after 10 h of treatment and could only be reduced to 45 nm after 12 h. A single-chamber self-oscillating oscillator proved that dual-chamber self-oscillating oscillator polishing is possible. Qianfa et al. [23] used the self-excited oscillating abrasive water jet method (SEO-AWJM) to polish silicon nitride materials and fixed-point treatment for 10 min. Based on these results, the maximum abduction depth of the SEO-AWJM was 9.4 μm. The workpiece surface roughness decreased from 108.9 nm to approximately 51.3 nm.

In summary, researchers have conducted various studies on the generation mechanism of fluid self-excitation, structural parameters of the chamber, and influencing factors of the self-excitation effect, which provide a basis for applying self-excited oscillations. Furthermore, fluid self-excited pulse characteristics can enhance the instantaneous velocity of the jet. Therefore, it is feasible to use a self-excited fluid to improve the processing efficiency of the abrasive jet. However, there is a lack of relevant research on the removal characteristics of self-excited oscillating pulsed abrasive waterjets, particularly on the influence of the process parameters on the erosion morphology and micropit parameters on the material surface.

In this paper, we first introduce the basic principle of a self-excited oscillating pulsed abrasive water jet (SEO-AWJ) and the mechanism of material removal during polishing. Then, we analyze the flow field characteristics using ANSYS Fluent software to determine the optimal scheme cavity length. Subsequently, we analyze the optimal cavity length under different external flow field structures. Finally, a numerical simulation of material erosion was conducted, material removal experiments were conducted below the optimal length of the self-excited oscillation cavity, and the influence of relevant process parameters on the erosion morphology and erosion depth of SiC polishing was analyzed.

## 2. Simulation

### 2.1. Fluid Control and Erosion Model

Ansys Fluent 18.0 was used to compute and analyze the erosion model of the self-excited oscillating abrasive jet of water. The overall model consisted of two parts: the internal flow field of the self-excited oscillation chamber and the external flow field impinging on the workpiece. A mixed multiphase flow model was chosen to simulate a three-phase gas-liquid-solid flow. Since there are many vortices in the self-excited oscillating cavity and this simulation focuses on near-wall erosion, the turbulence model adopts the realizable k-ε model. The model suits the prototypical jet flow, high curvature, and strong vortex shedding [24]. Its computation is relatively accurate when simulating flow near the wall and is well-suited to adverse pressure gradients, consistent with research on this subject.

The incompressible fluid used in this simulation was mean water, and its continuity equation is expressed as

(1) $$\frac{\partial u_{i}}{\partial x_{i}}=0,i=1,2,3.$$

The momentum equation is

(2) $$\frac{\partial {\bar{v}}_{i}}{\partial t}+\frac{\partial {\bar{v}}_{i}{\bar{v}}_{j}}{\partial x_{j}}=-\frac{1}{\rho }\frac{\partial \bar{p}}{\partial x_{i}}+\sigma \frac{\partial^{2}{\bar{v}}_{i}}{\partial x_{i}\partial x_{j}}-\frac{\partial {\bar{\tau }}_{ij}}{\partial x_{j}}$$

Here, ρ and *p* are the density and pressure, respectively; “-” represents the variable filtered at the grid level; ${\bar{v}}_{i}$ is the instantaneous velocity; $x_{i}$ is the three-dimensional coordinate direction; *i* is the spatial dimension of the tensor, *j* is the time dimension of the tensor; and ${\bar{\tau }}_{ij}$ is the modified subgrid scale tensor.

Fluent provides a general erosion rate calculation model [25]:

(3) $$R_{erosion}=\sum_{p=1}^{N_{particles}}\frac{m_{a}C\left(d_{a}\right)f\left(\theta \right)v^{b\left(v\right)}}{S_{face}}$$

Here, *R_erosion_* is the erosion rate, kg/(m^2^∙s); *N_particles_* is the number of particles; *m_a_* is the particle mass flow rate, kg/s; *C*(*d_a_*) is the particle size function, which is taken as 1.8 × 10^−9^; *f*(*θ*) is the impact angle function, *v* is the particle impact velocity, m/s, *b(v)* is 2.6; *S_face_* is the area of the processed wall, m^2^.

### 2.2. Simulation Physical Model and Boundary Setting

**Figure 1:**
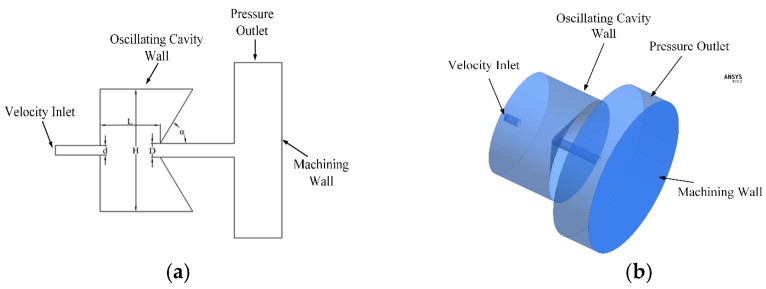
Self-excited oscillating cavity structure and boundary setting. Two-dimensional cavity structure (**a**). Three-dimensional boundary conditions (**b**).

**Figure 2:**
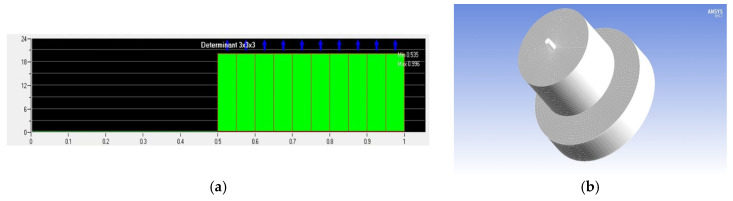
Three-dimensional grid sketch and grid quality diagram. Grid quality diagram (**a**). Three-dimensional grid sketch (**b**).

The simulation investigated the erosion of the treated wall surface in an external flow field and used the ICEM ANSYS software for mesh modeling and splitting. First, a two-dimensional cross-sectional view of the cavity is shown in Figure 1, and the specific structural parameters are listed in Table 1. Next, the structural grid is split and activated using the type of method. Considering the near-wall effect, the grids near the oscillation cavity and the processing wall are all dense, and the overall split grid model quality is greater than 0.5 (Figure 2). After the grid division is complete, the fluidic software ANSYS is used to simulate the treatment flow field, and the key parameters are fixed (Table 2).

Because the abrasive particles are displaced by the fluid medium, the discrete phase model must be started simultaneously when calculating the continuous phase. The erosion option must be activated, as with the solution settings, and the SIMPLE coupling method for the discrete pressure-velocity-pressure format is employed using PRESTO! (mainly used in high swirling flows and rapid pressure change flows) and second order for other parameters. Like the fluid simulation, the abraded particle phase uses the velocity input as the input boundary. This velocity was consistent with that of the fluid. The inlet was at atmospheric pressure, and the outlet boundary was at atmospheric pressure. All walls employed fixed no-slip boundary conditions. The type of wall–abrasive particle collision was set to be reflected. The results were imported into CFD-POST for post-processing analyses at the end of the calculation.

## 3. Material and Experiment

### 3.1. Materials and Pretreatment

**Figure 3:**
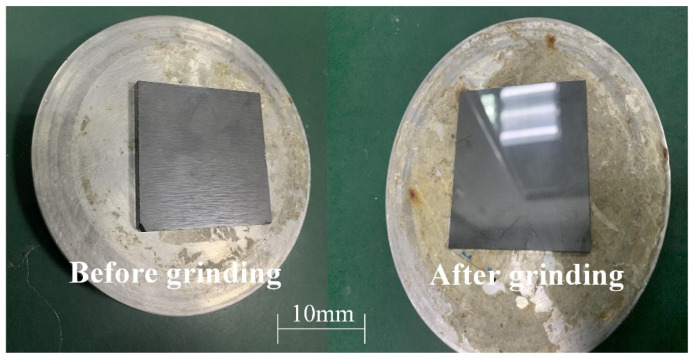
Silicon carbide substrate before and after grinding.

In this experiment, the workpiece material was a SiC substrate, the abraded material was SiC particles, and the polishing fluid consisted of deionized water, SiC-abraded particles, and dispersants. Depending on the requirements of the experiment, this study takes a silicon carbide substrate as the treatment object to investigate the influence of the self-excited momentum characteristics of the fluid on the treatment effect. The experiment measured the 3D surface profile and depth of removal of the treated workpiece and compared it to data measured after traditional abrasive water-jet processing. First, the original workpiece surface must be ensured to be relatively flat. All the samples were coarsely ground using a flat-grinding machine before processing. Images before and after grinding the SiC substrate are shown in Figure 3. From the figure, there are apparent scratch-like processing traces on the surface of the substrate before grinding, almost no mirror effect, and the quality of the surface does not match the experimental requirements. However, after grinding with 320# silicon carbide powder, the previous traces disappeared, with only a few scratches, and the effect of the workpiece mirror surface was evident. Randomly taking five points on the surface to be measured, the mean value was obtained, and the Ra surface roughness of the milled silicon carbide substrate was 30 nm.

### 3.2. SEO-AWJ Experiment Platform

**Figure 4:**
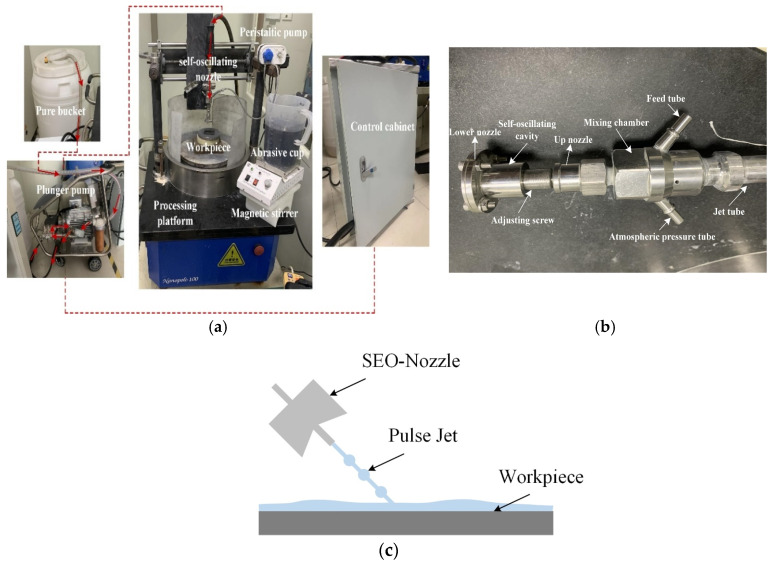
Self-excited oscillating abrasive water jet polishing device. The physical image of the experimental platform (**a**). Self-excited oscillating nozzle (**b**). Schematic illustration of SEO-AWJM (**c**).

Figure 4a shows the experimental self-excited oscillating abrasive waterjet platform. The main components of this experimental platform were a high-pressure piston pump, an abrasive pump, a self-excited oscillating nozzle device, a pure water bucket, and a frequency converter control cabinet. The performance of the piston pump directly influences the processability and efficiency of a jet engine. A high-pressure plunger pump with an output pressure of 50 Mpa is chosen. Pure water was pressurized to a preset pressure using a high-pressure piston pump to stabilize the energy. The abrasive pump simultaneously pumps the abrasive slurry continuously into the mixing chamber so that pure water and abrasive slurry are completely mixed in the mixing chamber to form a high-pressure abrasive jet of water, which then enters the self-excited oscillation chamber to be modulated. The self-excited oscillating abrasive jet is the heart of the experimental platform, consisting of two parts (i.e., the abrasive mixing chamber and the self-excited oscillation chamber), as shown in Figure 4b. The high-speed jet is mixed uniformly with the abrasive slurry in the mixing chamber to form a polishing fluid. The continuous jet of fluid is converted to a pulsed jet using the unique structure and boundary conditions of the self-excited oscillating cavity. The schematic illustration of SEO-AWJM is shown in Figure 4c. All the self-excited oscillating nozzles are attached to the treatment spindle, which can adjust the arbitrary target distance and treatment angle. The length of the self-excited oscillating cavity can be arbitrarily tuned to achieve self-excited pulsed oscillating abrasive water jets with different output tip speeds.

### 3.3. Experimental Method

During the self-excited oscillating pulsed abrasive water jet erosion process, the experiment selected the jet pressure, standoff, and treatment angle as the single-factor process parameters affecting erosion quality. In addition, a polished SiC comparison experiment was performed without the self-excited oscillating cavity structure. Thirty sets of experimental material removal data were acquired, and the specific parameter settings are listed in Table 3.

The treated workpiece was attached to the platform and perpendicular to the jet. A target distance of 10 mm was maintained, and the concentration of the abrasive material was maintained at 5%. The silicon carbide wafer was eroded at fixed points during the experiment, and the erosion time for a single experiment was 10 min. At the end of the experiment, a KEYENCE super depth-of-field microscope was used to observe the erosion morphology and acquired workpiece surface depth data.

## 4. Results and Discussion

### 4.1. Simulation Results

#### 4.1.1. Analysis of Processing Flow Field

A 2D grid model and 2D simulation solver were used to simulate the entire treatment flow field for ease of computation and to discuss the variation in the axial velocity under different cavity lengths.

**Figure 5:**
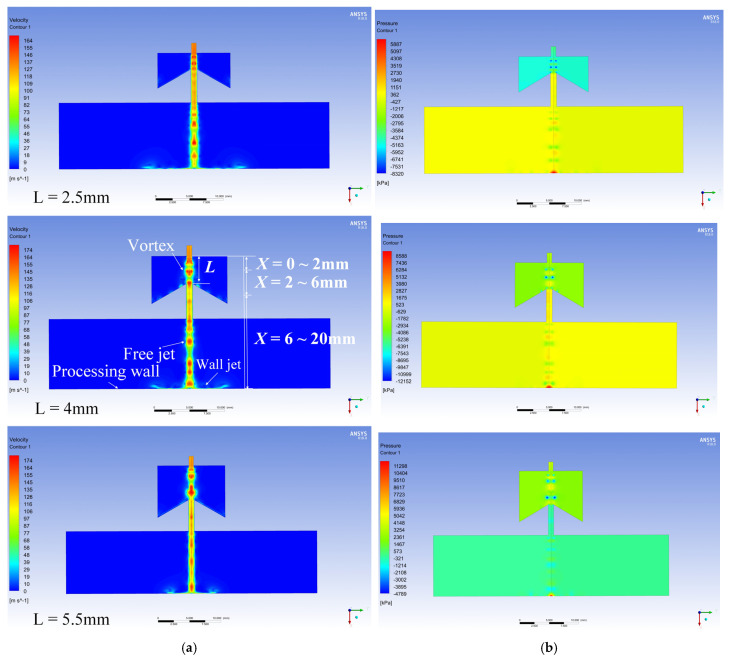
Flow field velocity and pressure contour under three cavity lengths. Velocity contour (**a**). Pressure contour (**b**).

A three-cavity length flow field simulation was performed under the same setup conditions: a vertical jet was maintained, the impact distance S was 10 mm, the particle mass flow rate M was 6.7 × 10^−4^ kg·s^−1^, the particle size D was 5 μm, and the inlet speed V was 135 m/s. In conventional jet impingement processing, air resistance is ignored, and the simulated velocity contour pressure. Figure 5a shows the image of the cloud. The figure shows that the behavior of the fluid motion below the three cavity lengths was fundamentally the same. As the jet beam enters the cavity of the self-excited oscillations, it interacts with the unstable shear layer of the cavity. As a result, a set of discontinuous eddy current perturbations form in the axial direction and are subsequently affected by the viscosity and shearing of the surrounding fluid. They continue to grow downstream of the chamber and collide with the conical surface downstream to generate upward pressure perturbations, propagating and inducing new perturbations upstream and forming periodic perturbations. From the exit of the self-excited oscillation chamber, the continuous jet formed an impulsive jet. The velocity pulse chambers left the oscillation chamber and continued to flow downstream along the axis before entering the external flow field of the treatment.

After the abrasive fluid was sprayed out of the chamber, it diverged to either side along the central axis of the jet, and the velocity attenuation was significant. An area of high-pressure forms at the impact point of the treatment wall, where the static pressure is the highest and the velocity is the lowest, referred to as the stagnation point.

The pressure cloud diagram in Figure 5b shows the red high-pressure zone formed at the machining center. By combining this with the velocity cloud diagram, it can be observed that the flow from the wall jet is promoted to flow on both sides under the action of the pressure difference between the static and wall jet surfaces. As a result, the velocity of the wall jet gradually increased from the center to either side. As the tangential distance increased, the jet’s kinetic energy attenuated, and the velocity started to decrease. The wall jet velocity was distributed in an inverted bell shape across the simulation domain.

**Figure 6:**
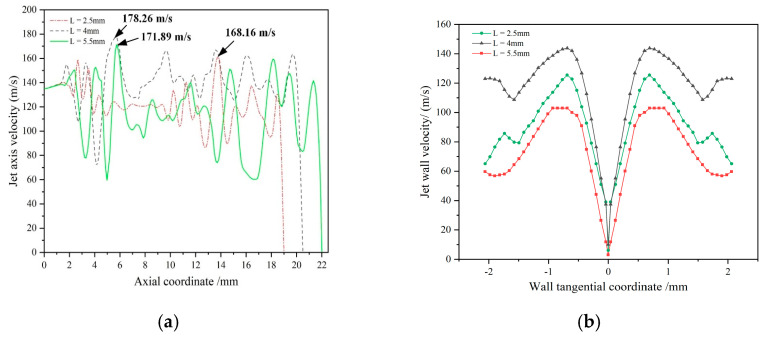
Jet axial and wall velocity curves under three cavity lengths. Axis velocity (**a**). Processing wall velocity (**b**).

The axial velocity distribution curves of the jet and treatment wall for the three different chamber lengths are shown in Figure 6. As the jet has been accelerated by the self-excited oscillation chamber and has transitioned from a steady jet to a pulsating jet, it shows one after the other velocity pulse, the axial velocity develops in a waveform along the axial distance, and the magnitude of the velocity fluctuation is different under different cavity lengths. Considering the analysis of the axial velocity curve when L = 4 mm, as shown in Figure 5 and Figure 6, when the axial distance X = 0–2 mm at an initial flow velocity of 135 m/s, the jet had just reached the top nozzle of the chamber and had not yet entered the chamber. The difference in velocity for the three-chamber lengths was insignificant at this time, and they were all continuous jets. In this figure, the curve is a continuous straight line. As shown in Figure 5 and Figure 6, at X = 2–6 mm, the jet entered the self-excited oscillation chamber, the main stage of jet acceleration. As shown in the figure, the velocity curve changed from a continuous straight line to a wavy line with perturbations at the top and bottom, which demonstrates that the self-excited oscillating chamber formed a large number of velocity fluctuations, and the jet completed the conversion from a continuous jet to a pulsed one. When L = 4 mm, the peak velocity was 178.26 m/s. When X > 6 mm, the jet flow entered the velocity decay stage from the acceleration stage of the pulse; however, there were still significant fluctuations. As the jet beam moved toward the wall, owing to a stagnation region, the velocity of the jet rapidly decreased, approaching zero. Then, the jet was forced to flow along the edges on either side of the wall to form a wall jet, and the velocity temporarily increased. As the travel distance increased, the kinetic energy of the jet decreased, and the velocity gradually approached zero.

#### 4.1.2. Effect of Impact Distance on Wall Erosion

**Figure 7:**
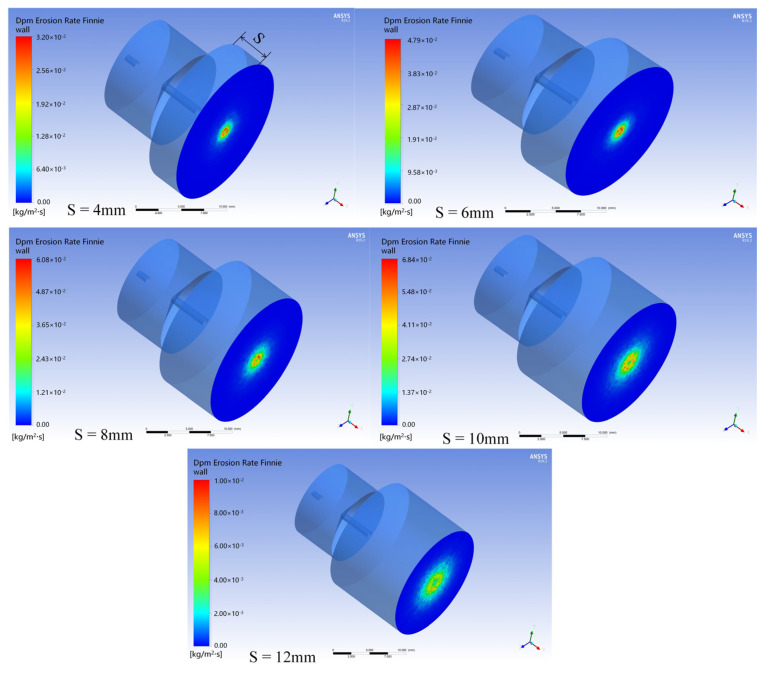
Wall erosion distribution cloud by impact distance.

At *L* = 4 mm, the self-excited oscillating abrasive jet of water eroded the workpiece wall to suppress the shape at different target distances under vertical incidence conditions. The influence of impact distance on the results of treatment wall erosion is investigated for an inlet velocity *V* of 135 m/s, the particle mass flow rate M was 6.7 × 10^−4^ kg·s^−1^, the particle size D was 5 μm, and the cloud pattern of erosion distribution is shown in Figure 7, when the cavity length and other parameters are constant, there is no linear relationship between the treated wall erosion rate and the target distance. However, there is a relationship between erosion and the optimal target distance for the maximum eclipse rate.

The jet beam was divided into transition, basic, and dissipative sections based on the basic characteristics of the jet beam structure [26]. Moreover, S = 4 mm corresponds to the transitional section of the jet stream. Then, the jet flow accelerates through the self-excited oscillating cavity, enters the external flow field, and moves farther into the vicinity of the treatment wall. Based on the image of the erosional cloud, once it impacts the wall surface, the range is relatively concentrated, which is an evidence that the jet beam at this epoch is relatively compact, has some capacity for processing, and is in the tendency to change to the basic segment. At S = 6–8 mm, the jet is in the baseline segment, which is the primary acceleration of the jet phase. As the kinetic energy of the jet increases, the stronger the impact on the wall, the larger the corresponding erosion rate. The image of the erosional cloud shows that when S = 8 mm, the range of erosion is wider than that of the previous two, indicating that the jet improves the jet beam and diverges while striking. When S = 10 mm, the flow of the jet accelerated to its maximum value, and the shock on the wall was the most intense. Once the jet flow reaches the tip and tail of the baseline section, it enters the dissipative section. From the cloud map, it can also be observed that, for the wall surface at this time, the range of impact is much larger than the first three, and the wall erosion rate reaches the maximum value. When the jet beam develops further to S = 12 mm, the jet beam is already in the dissipative phase. If the kinetic energy loss from the jet is severe, the velocity attenuation is evident, the wall erosion rate decreases, and the erosional range further widens. Subsequently, the jet beam lacked processing capability as the shock distance increased.

**Figure 8:**
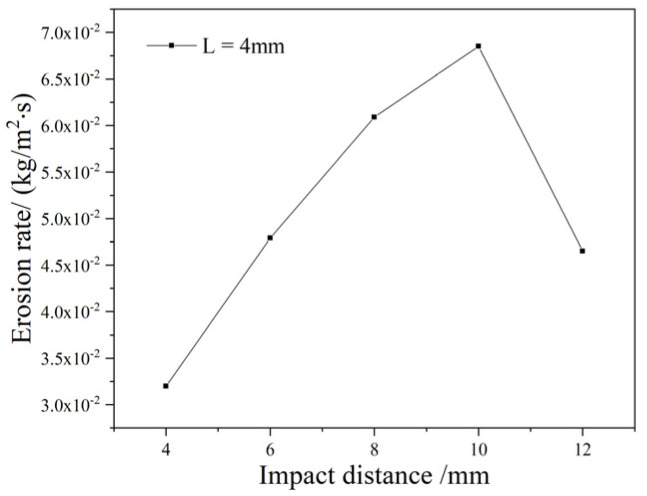
Influence curve with impact distance.

The influence curve for the erosion rate of the treated wall is shown in Figure 8. The wall erosion rate increases first and decreases as the impact distance increases. If the optimal impact distance S is 10 mm, the maximum value of the wall erosion rate is 0.068 kg/(m^2^·s), which has been previously analyzed. However, when S = 12 mm, the jet flow was already within the dissipative section, and the velocity could still maintain a certain magnitude while attenuating because of the inertia of the fluid. The graph shows that the erosion rate when S was 12 mm was larger than when S was 4 mm.

**Figure 9:**
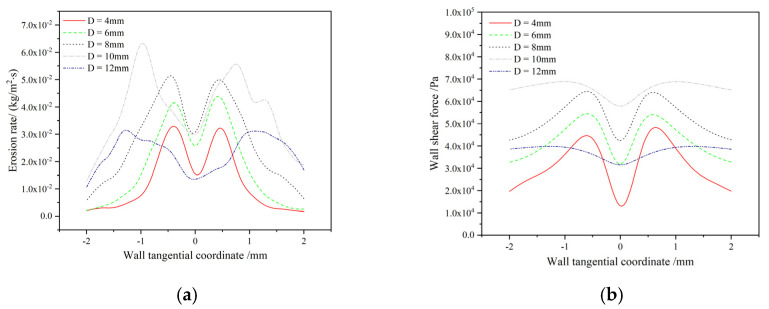
Simulation of workpiece wall removal under different impact distances. Wall erosion rate (**a**). Wall shear force (**b**).

When L = 4 mm, the erosion rate of the treated wall varies as a function of the tangential coordinates under different impact distances, as shown in Figure 9a. In general, regardless of the impact distance, the erosion rate of the wall increased from the center of the workpiece to either side. The tendency of the distribution to first increase and then decrease did not change, which is consistent with the changing law. When the shock distance is short, the interval between the peaks of the curve in the figure is relatively narrow, which matches the small erosional range of the previous erosional cloud image because the erosion rate of the wall increases from the machining center on both sides. Thus, the portion between the maximum erosion rate of the wall (corresponding to the peak of the curve) and the center of machining (corresponding to the curve’s trough) is the machining’s centre area. This was also the primary erosion zone of the material. The distance between the peaks in the curve also increases as the impact distance increases, and the range of erosion further increases. If the shock distance is considerably large, the impact capability of the jet is insufficient to remove material from the workpiece, and the curvature of the curve gradually flattens.

The shear force distribution on the workpiece wall at different impact distances is shown in Figure 9b. Like the wall erosion rate, the shear force distribution trend of the workpiece wall first increased and then decreased from the machining center surface outward. The existence of the stagnant layer causes a small portion of the particles to break through the stagnant layer to process the workpiece, and the majority of the remaining particles are impeded by the stagnant layer and travel along the edge of the machining center, which still has a high velocity; thus, the central edge region has a stronger cutting capacity. In other words, the shear force increases. The kinetic energy of the particles gradually decreased as the tangential distance further increased, and the shear force curve gradually approached zero as the particles almost no longer had processing capability.

#### 4.1.3. Effect of Impact Angle on Processing Wall Erosion

**Figure 10:**
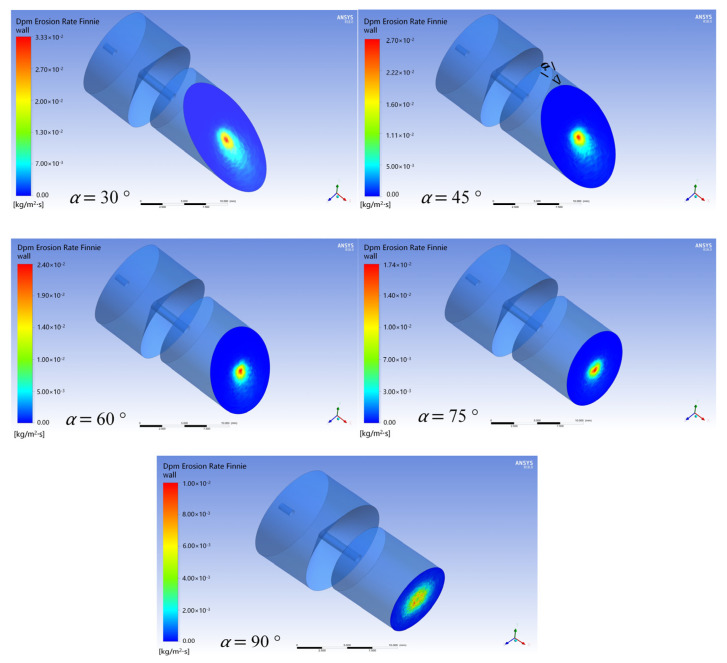
Processing Wall erosion distribution cloud by machining angle.

For the grid model, impact distances are kept fixed at 10 mm. The oscillating self-excited abrasive water jet erodes the workpiece wall to suppress the shape under different treatment angles below L = 4 mm, the particle mass flow rate M was 4.2 × 10^−4^ kg·s^−1^, the particle size D was 3 μm, and the inlet velocity V was 125 m/s. The influence of treatment angle on the treated wall erosion results is then investigated, and the cloud pattern of erosion distribution is shown in Figure 10.

**Figure 11:**
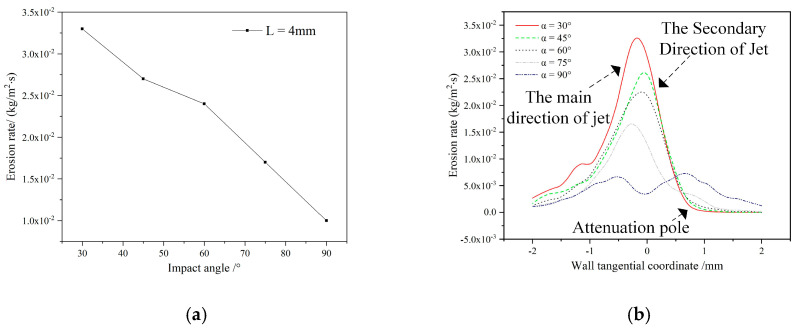
Simulation of workpiece wall removal under different impact distances. Influence curve of erosion rate (**a**). Wall erosion rate (**b**).

As shown in Figure 10, when the cavity length and other parameters are constant, there is a linear relationship between the erosion rate of the treated wall surface and the treatment angle, and the contour of the vertical treatment shows a tendency for the erosion rate to be low in the center and high on both sides. The situation is symmetric in the axis, similar to the previous sections. However, the erosion rate in the central region gradually increases as the angle gradually decreases, and the smaller the angle, the more asymmetric the erosional range. Moreover, the treatment zone mainly extends toward the direction of the jet motion. This may be explained by the fact that when the angle is smaller, the tangential force component is larger, and the effect of cutting on the surface is dominant. A stagnation zone exists because the rebounding abrasive grains block the motion of the incoming abrasive grains. In the small-angle treatment, the rebounding abrasive grains, if more particles bounce toward the edge of the jet, will reduce the blockage effect, and the surface of the machining center will be plowed by more particles so that the erosion rate in the central zone is high. As a result, a smaller angle was observed. Figure 11a shows the influence curve of the wall erosion rate. When the treatment angle was 30°, the wall erosion rate reached a maximum value of 0.033 kg/(m^2^·s).

When L = 4 mm, the erosion rate of the treated wall at different impact distances varied as a function of the tangential coordinates, as shown in Figure 11b. When combined with the analysis of the erosion cloud image, the negative direction of the tangential coordinates is the principal direction of jet development. For the small-angle shock, when the jet collides with the wall, its bulk moves primarily in the negative direction. A small portion moves in the positive direction corresponding to the secondary jet development direction. Along with the lowest erosion rate in the central vertical impact zone, the erosion rate in the center zone was not the lowest when machined at other angles. As shown in the figure, when machined at a small angle, due to the relatively low erosion rate of the treatment area under the main jet flow direction, the larger the processing area in the center zone and positive direction, and the smaller the angle, the more apparent the case. As previously explained, the tangential component of the force increases as the angle decreases. Moreover, the larger the component of the tangential force, the more severe the cutting action on the wall. Because of this effect and the influence of gravity, the smaller the angle of attack, the more the jet moves in the main development direction; therefore, the degree of attenuation of the wall erosion rate is more serious in the jet flow secondary development direction, and the erosion rate attenuation distance in this direction is longer.

### 4.2. Experiment Results

#### 4.2.1. Influence of Jet Pressure on Processing Erosion

**Figure 12:**
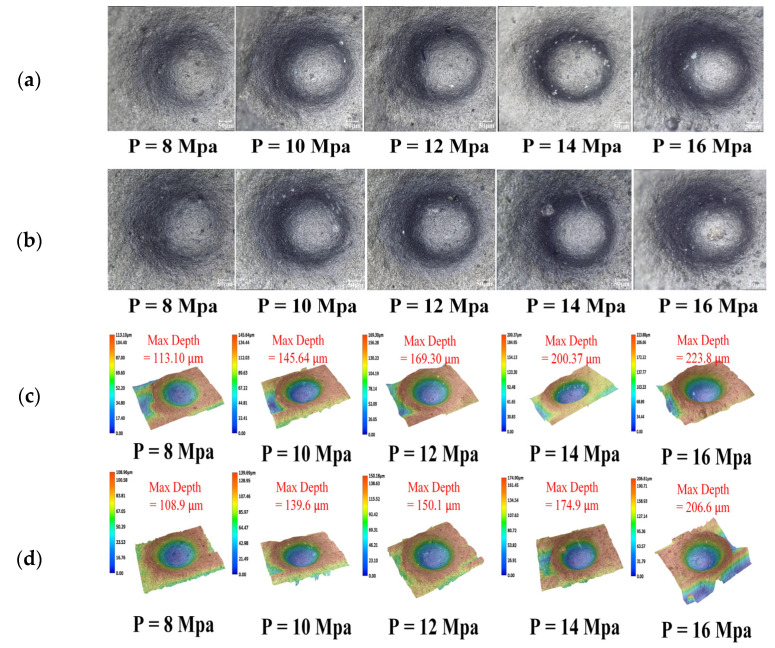
Erosion morphology and depth of cavities with and without self-excited oscillation under different jet pressures: (**a**) Erosion morphology of SEO-AWJM, (**b**) Erosion morphology of AWJM, (**c**) Erosion depth of SEO-AWJM, (**d**) Erosion depth of AWJM.

The single-factor experiment of jet pressure was performed when the cavity length was 4 mm, the incidence was vertical, and the standoff H was 10 mm. Table 3 lists the remaining parameters. Figure 12 shows the comparison of the erosional morphology and depth with and without a self-excited oscillating cavity structure.

As shown in Figure 12, the surface of the silicon carbide is eroded by the jet to form apparent pits, and the pits have a regular shape.

**Figure 13:**
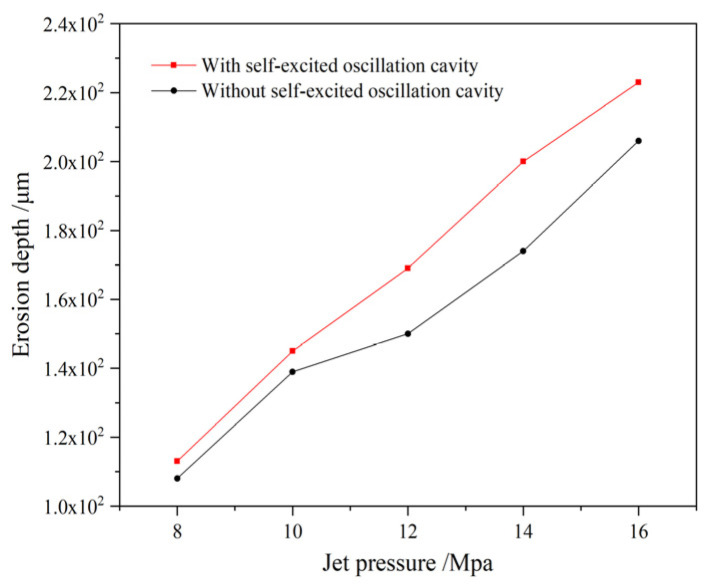
Erosion depth influence curve under different jet pressures.

Figure 13 shows the influence curve. The depth of erosion below the self-excited oscillating cavity structure was larger than that of traditional abrasive waterjet polishing. With a difference of up to 26 μm between the two depths, the depth of erosion increases, and the pressure of the jet increases up to 223 μm, which can be explained by the fact that the pressure energy of the water jet in the mixing chamber is converted to the kinetic energy of the abraded particles. Increasing the pressure of the jet directly increases the kinetic energy of the abraded particles, improving the erosion performance of a single abrasive particle (i.e., the ability to impact vertically and remove tangential shear on the surface of the material). This led to an increase in erosion depth. However, excessive water pressure increases the pressure on the wall of the self-excited oscillating cavity. The cavity is damaged when the wall pressure reaches the material strength limit; therefore, the velocity must also be controlled within an appropriate range.

#### 4.2.2. Influence of Standoff on Processing Erosion

**Figure 14:**
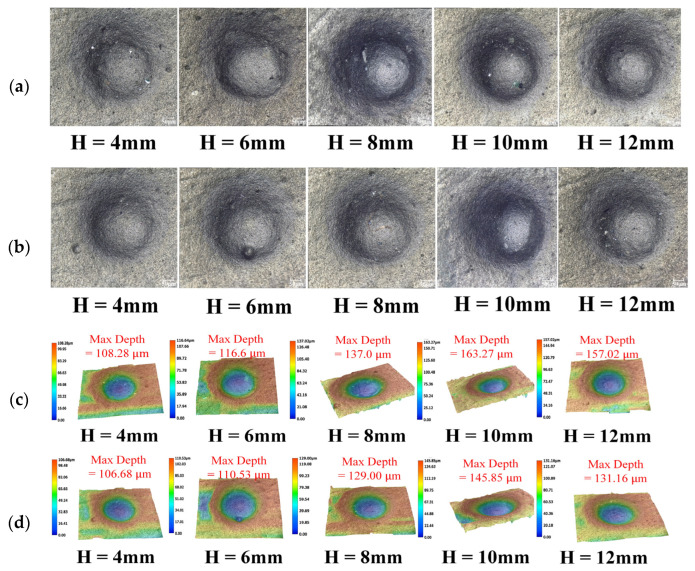
Erosion morphology and depth of cavities with and without self-excited oscillation under different standoffs: (**a**) Erosion morphology of SEO-AWJM, (**b**) Erosion morphology of AWJM, (**c**) Erosion depth of SEO-AWJM, (**d**) Erosion depth of AWJM.

The single-factor experiment of the standoff was performed when the cavity length was 4 mm, the incidence was vertical, and the jet pressure *p* was 12 mpa. Table 3 lists the remaining parameters. Figure 14 shows the comparison of the erosional morphology and depth with and without a self-excited oscillating cavity structure.

**Figure 15:**
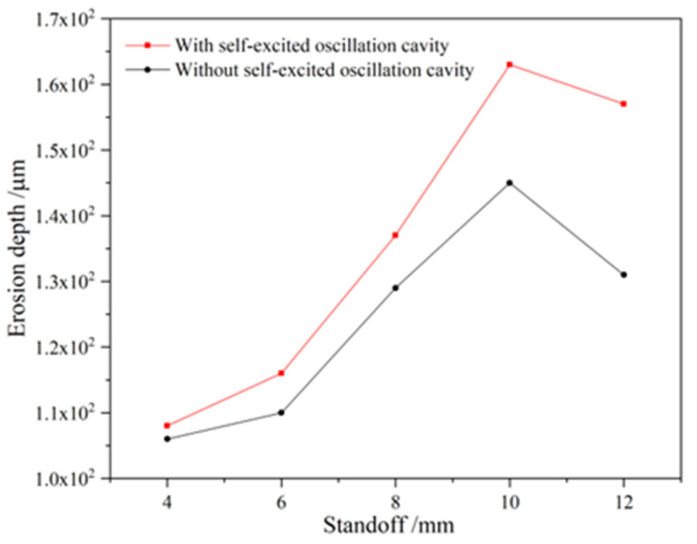
Erosion depth influences curves under different standoffs.

Figure 15 shows the influence curve. The depth of erosion below the self-excited oscillating cavity structure is larger than that of traditional abrasive water-jet polishing. The difference between the two depths is as much as 26 μm. In either case, there is an optimal target distance for erosion depth. This agrees with what has been mentioned in the simulation because the jet beam development is split into three stages: the transition stage, base stage, and dissipation section, only when the end of the base section is near the dissipation section since the kinetic energy of the jet is allowed to reach the maximum value. When the target distance is between 4 and 10 mm, the impact force on the workpiece surface will be larger as the jet’s kinetic energy always increases. The depth of abduction has a linear relationship to the target distance, and the jet beam enters the dissipation section when the target distance is greater than or equal to 12 mm. Since the kinetic energy of the jet flow has started to decay, the impact force on the workpiece wall also decreases, which is insufficient for the material to produce efficient removal. Therefore, the optimal value of the target distance should be chosen to improve processing efficiency.

#### 4.2.3. Influence of Processing Angle on Processing Erosion

**Figure 16:**
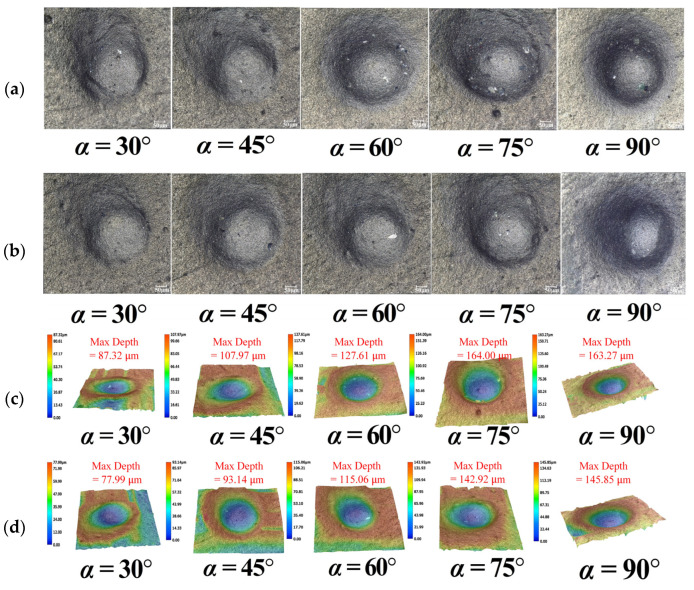
Erosion morphology and depth of cavities with and without self-excited oscillation under different impact angles: (**a**) Erosion morphology of SEO-AWJM, (**b**) Erosion morphology of AWJM, (**c**) Erosion depth of SEO-AWJM, (**d**) Erosion depth of AWJM.

A single-factor experiment of the processing angle was conducted when the cavity length was 4 mm, the jet pressure *p* was 12 Mpa, and the standoff H was 10 mm. The other parameters are listed in Table 3. Figure 16 shows the erosion morphology and depth map with and without the self-excited oscillation cavity structure.

**Figure 17:**
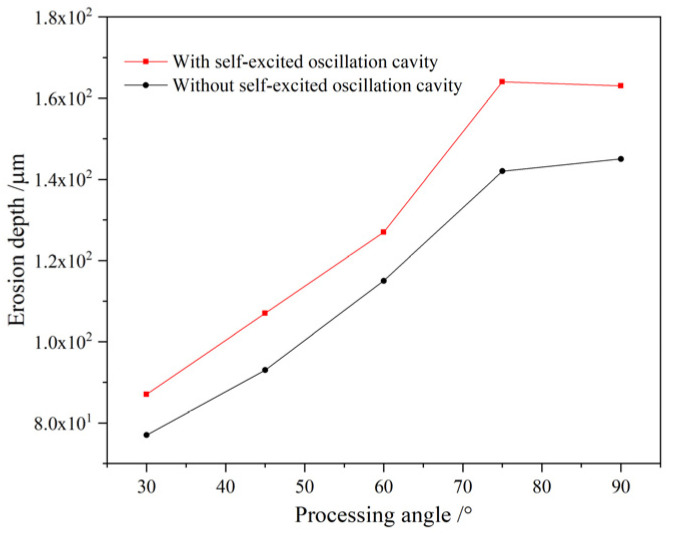
Erosion depth influence curve under different angles.

Figure 17 shows the influence curve. The depth of erosion with the self-excited oscillating cavity structure is larger than that of traditional abrasive water-jet polishing, and the difference in depth between the two is as much as 22 μm. This value can be as high as 164 μm. The depth of erosion gradually increased with the treatment angle. This contrasts the phenomenon in which the erosion rate linearly decreases as the angle increases, as described in the simulation in Section 4.1.3. The force exerted by the abrasive particles entrained by the jet beam on the wall of the material is split into the vertical impact force and transverse shear force, and the depth of erosion is determined by the vertical impact force produced. The transverse shear force played a dominant role when the treatment angle was 30°. The primary method for removing the workpiece surface is plowing, and the depth of erosion is low. As the angle increased from 30° to 75°, the vertical proportion of the shock component became increasingly large, and brittle fracture of the surface of the material was dominant; thus, the depth of removal was larger. As the angle approaches the vertical direction, the abduction depth approaches a critical value and does not change.

## 5. Conclusions

In this study, a self-oscillating pulsed abrasive waterjet polishing experiment was conducted on silicon carbide, and the material removal characteristics were investigated. First, a numerical simulation was carried out to analyze the flow field characteristics of the self-excited oscillation cavity, such as axial and wall surface velocities, to determine the optimal cavity length. Then, we discussed the influence of the external flow field structure on the surface erosion rate of the workpiece material. Then, through the self-excited oscillation with or without fluid, an experiment for the characteristic abrasive water jet polishing silicon carbide material was conducted to verify the influence of process parameter changes on the micro-pit morphology and erosion depth of the workpiece. The main conclusions are as follows.

A simulation of the self-excited oscillating abrasive water jet erosion determined that when the cavity length was 4 mm, the axial velocity of the entire flow field decayed the slowest, and the peak axial velocity was the largest (up to 178.26 m/s). The maximum erosion rate varied linearly with the impact angle, and the impact distance had an optimal value that maximized the erosion rate. The erosion rate was the highest when the impact distance was 10 mm.

Through an abrasive water jet polishing experiment with/without a self-excited oscillating cavity, by comparing the depth of the micropit on the surface of the processed material, it was found that the self-excited oscillating pulse abrasive water jet can cause a greater removal depth than an ordinary abrasive water jet in the same time, reaching a maximum of 26 microns.

The influences of the relevant process parameters on the erosion depth were investigated, and the experimental results were consistent with the simulation results. The increase in the jet pressure and machining angle increased the erosion depth. However, because the erosion rate and depth on the surface of the workpiece are mainly determined by the tangential shear force and vertical impact force, rather than the processing distance and processing angle, the jet pressure directly affects the kinetic energy of the jet, influencing the corrosion depth. When there is a self-excited oscillation cavity, the maximum erosion depth of the workpiece surface can reach 223 μm.

SEO-AWJM has good research value and application prospects in the field of precision machining since it can replace abrasive water jets in processing free-form surfaces, complex three-dimensional surfaces, smooth surfaces, and tiny inner cavities, thereby improving processing efficiency. However, it has shortcomings, such as low machining efficiency, complex structure, insufficient stability, and serious nozzle wear. Therefore, it is necessary to explore further the process parameters, removal function model, and processing path optimization to improve processing accuracy and efficiency.

## Figures and Tables

**Table 1:** Main structural parameters of the self-excited oscillation cavity [23].

Parameter	Value
Lower nozzle diameter/Upper nozzle diameter (D/d)	1.32
Chamber diameter/Upper nozzle diameter (H/d)	5.92
Cavity length L (mm)	2.5, 4, 5.5
Collision wall angle α (°)	60
Upper nozzle diameter d (mm)	0.76

**Table 2:** The main parameters of the simulation.

Parameter	Value
Water density (kg·m^−3^)	998
Gravitational acceleration (m·s^−2^)	9.8
Particle density (kg·m^−3^)	3515
Inlet velocity V (m/s) Impact distance S (mm) Particle mass flow rate M (kg·s^−1^) Particle size D (μm)	125,135 4~10 4.2 × 10^−4^, 6.7 × 10^−4^ 3, 5
Impact angle α (°)	30~90
Hydraulic diameter (mm)	55
Steady-state steps	3000
Turbulence Model	Realizable k-ε
Multiphase Flow Model	Mixture

**Table 3:** Processing parameters of self-excited oscillating abrasive water jet.

Jet Pressure *p*/(Mpa)	Particle Size /(#)	Standoff H/(mm)	Processing Angle α/(°)	Abrasive Concentration /(wt%)	Abrasive Flow /(mL/min)	Processing Time/(min)	Workpiece
8, 10, 12, 14, 16	1000 (SiC)	4, 6, 8, 10, 12	30°, 45°, 60°, 75°, 90°	5	50	10	SiC wafer

## Data Availability

Data are only available upon request due to restrictions regarding privacy and ethics. Data presented in this study are available from the corresponding author upon request. The data are not publicly available because of relationships with other ongoing studies.
